# The Impact and Feasibility of Introducing Height-Adjustable Desks on Adolescents' Sitting in a Secondary School Classroom

**DOI:** 10.3934/publichealth.2016.2.274

**Published:** 2016-05-11

**Authors:** Bronwyn Sudholz, Anna Timperio, Nicola D. Ridgers, David W. Dunstan, Rick Baldock, Bernie Holland, Jo Salmon

**Affiliations:** 1 Deakin University, Geelong, Australia. Institute for Physical Activity and Nutrition (IPAN), School of Exercise and Nutrition Sciences; 2 Baker IDI Heart and Diabetes Institute, Melbourne, VIC, Australia; 3 School of Public Health, The University of Queensland, Herston Road, Herston, Brisbane, QLD 4006, Australia; 4 Department of Epidemiology and Preventive Medicine, Monash University, Malvern East, VIC, Australia; 5 School of Sport Science, Exercise and Health, The University of Western Australia, Perth, WA, Australia; 6 Mary MacKillop Institute for Health Research, The Australian Catholic University, Melbourne, VIC, Australia; 7 The Australian Council for Health and Physical Education, South Australian Branch, SA, Australia; 8 The Australian Council for Health and Physical Education, Victorian Branch, SA, Australia

**Keywords:** *activ*PAL, ActiGraph, sedentary behaviour, classroom sitting, adolescents, school

## Abstract

Children spend over 60% of their school day sitting; much of this occurs in the classroom. Emerging research has examined the impact of environmental interventions on classroom sitting. While this research is promising, it has predominantly focused on the primary school setting. This study examined the impact and feasibility of height-adjustable desks on time spent sitting/standing during classroom lessons in a secondary school. Traditional desks in a Melbourne secondary school classroom were replaced with 27 height-adjustable desks (intervention classroom). Forty-three adolescents (51% male; mean age 13.7 ± 1.4 years) from Grades 7, 9 and 10 wore an inclinometer and accelerometer for schooldays and completed a survey after using the desks during lessons for seven weeks. Ten teachers (50% male) completed a survey. Time spent sitting, standing, and the length of sitting bouts were compared between periods when adolescents were in the intervention classroom versus traditional classrooms (matched on teacher and subject). Compared to the traditional classroom, adolescents spent 25% less time sitting and 24% more time standing in the intervention classroom (effect size > 0.8), and had a greater frequency of short sitting bouts and fewer longer bouts. The majority of teachers (71%) and students (70%) reported wanting to continue to use the height-adjustable desks. When standing during lessons, adolescents reported working well (69%); however, a third reported difficulties paying attention (28%) and becoming distracted (36%). Few teachers reported negative influences on adolescents' ability to work (14%) and concentrate (14%). Half the adolescents reported leg, or back pain with standing. Introducing height-adjustable desks resulted in lower levels of sitting compared with traditional classrooms, was acceptable and had some adverse effects on concentration and discomfort. The study provides preliminary evidence that height-adjustable desks may help reduce prolonged sitting in school among adolescents. Future research should incorporate a control group and explore behavioural and academic outcomes.

## 1. Introduction

Excessive sedentary behaviour (sitting while expending ≤ 1.5 metabolic equivalent units [METs] of rest) is now recognised as an independent health risk factor [1]. Among youth (children 5–12 years; adolescents 13–17 years), sedentary behaviour has been adversely associated with cardio-metabolic biomarkers, adiposity markers, fitness, cognitive development and academic achievement [2]–[6]. Specific sedentary behaviours (i.e. screen time) among children and adolescents have been found to track into adulthood [7] and are associated with reduced physical and psychosocial health [4], [8], [9]. Consequently, reducing the amount of time youth spend sitting is likely to be important for immediate and long-term health. Several countries have now incorporated explicit sedentary behaviour targets into their national guidelines [10]–[12]. Australian recommendations state that children (5–12 years) and young people (13–17 years) should minimise the time they spend sedentary every day and break up long periods of sitting as much as possible [13].

Recent studies using objective measures (inclinometers) have shown that children spend more than 60% of school time sitting, approximately 70% of class time sitting [14], [15] and that sedentary time (assessed with accelerometers) increases from childhood to adolescence [16]. Furthermore, prevalence data (using inclinometers) indicates adolescent females spend approximately 10 waking hours per day in sedentary activities [17], and the school setting is associated with a greater frequency of prolonged sitting bouts (> 20 minutes) compare to after school hours [18]. In light of emerging health research and the high proportion of sitting time in the school context, researchers have begun to explore the effects of environmental interventions on classroom sitting [19], [20]. To date, this research has typically removed traditional desks and chairs from classrooms and replaced them with active work desks that are either height-adjustable (i.e. can be easily adjusted from a sitting to standing height), or stand-biased (i.e. require adolescents to stand when using) in primary (elementary) schools [19], [20]. In these studies, irrespective of the type of active workstation (height-adjustable or stand-biased), sitting has been found to reduce and standing time increase (across the whole school day, or during class time), relative to controls or when compared to traditional classroom desks [17], [21]–[23]. Furthermore, several studies have reported increases in energy expenditure [24]–[26], benefits [27], [28] or no change [22] in musculoskeletal outcomes, and improvements or no change (i.e. no detrimental effect) in academic-related behaviours [23], [29]–[31].

While these results are favourable, this research is predominantly limited to children within the primary (elementary) school setting [19], [20]. Few studies have examined the effects of height-adjustable desks in the secondary school setting on adolescents' sitting time [27]. One potential reason for this may be due to the structure of the school day, where adolescents typically have lessons in different classrooms throughout the day depending on the subjects being taught by different teachers. This may impact on the potential effects of height-adjustable desks on sitting time in the secondary school setting due to more limited intervention exposure (unless a school can outfit the entire school with such desks). To date, only one study has examined the impact of height-adjustable desks on musculoskeletal and academic outcomes in a secondary school setting, wherein favourable outcomes (e.g. improved posture and strength, and overall grades) were found among students using the height-adjustable desks relative to control students [27]. There have been no published studies investigating the impact of height-adjustable desks in the secondary school setting on adolescents' sitting time in class.

This pilot study aimed to examine the impact (on classroom sitting, sitting bouts, standing, light-intensity physical activity, and academic behaviours) and feasibility (from the perspective of adolescents and teachers) of introducing height-adjustable desks in one secondary school classroom.

## 2. Methods

The study used a within-subject cross-sectional design, which involved equipping one classroom within a government secondary school (public high school) in Melbourne, Australia with 27 height-adjustable desks (i.e. one desk for each student and the teacher; Ergotron LearnFit® Adjustable Standing Desk, Sydney, Australia) and large backless laboratory stools (Furnware Bodyfurn Lab stool, Melbourne, Australia) for seven weeks (August to October 2014). In this classroom, no specific daily sitting or standing targets were prescribed, nor were any professional development provided for teachers or information to the adolescents pertaining to the health effects of sitting.

Teachers (n = 17) and adolescents (six different classes ∼156 adolescents) who used the classroom equipped with height-adjustable desks were invited to participate in the evaluation component of the study, which took place after a period of familiarisation with the height-adjustable desks (seven weeks). Adolescents in the classes were provided with a brief introduction to what participation entailed and were provided with parental consent forms to take home and complete if they were interested. Adolescents wore an ActiGraph accelerometer and *activ*PAL inclinometer for five consecutive school days, and completed a self-report survey during class time when the monitors were collected. Adolescents were instructed to remove devices for water-based activities (i.e. swimming, showering). A monitor log book was provided for adolescents to record the type and duration of activities performed during any period in which the monitors were removed. School absence records were used to identify adolescents' school and non-school days. School timetables were used to identify when adolescents were using the classroom with the height-adjustable desks and when they were in a traditional classroom for the same subject with the same teacher, which served as a comparison lesson. On average, lessons lasted 69 minutes and there were three lessons per relevant subject per week. During the week adolescents wore the monitors, 51% (n = 22) had two lessons and 49% (n = 21) had one lesson in the classroom with the height-adjustable desks. The remaining lesson(s) for the relevant subject(s) occurred in traditional classrooms.

Teachers were sent an email summarising the components of an online survey, and if they were interested were invited to follow the link to the plain language statement. Teachers who agreed to the online consent form were directed to the online survey. Overall, 10 teachers (59% response rate), and 43 adolescents (28% response rate; 65% in Year 7) consented to participate. As it is an ethics requirement in Australia for active informed consent to be provided, no information was obtained concerning non-responders. Approval was received from Deakin University Human Ethics Advisory Group (Health) and the Department of Education and Early Childhood Development.

## 3. Measures

### 3.1. Classroom sitting, standing, and breaks in sitting time

The thigh-mounted activPAL3C inclinometer (Pal Technologies Ltd, Glasgow, UK) and hip-mounted ActiGraph GT3X+ accelerometer (Pensacola, FL, USA) were used to determine time spent sitting/sedentary, standing/light-intensity physical activity), and length of sitting and standing bouts during periods in which adolescents were in the classroom with the height-adjustable desk and comparison periods for the same subject/teacher. Adolescents were fitted with both devices and wore them during waking hours for five consecutive school days. Both devices have demonstrated reliability and validity for use in free-living studies [14], [32]. The ActiGraph was included in addition to the *activ*PAL (which examines changes in posture) as it is commonly used to assess changes in sedentary time in interventions, and enabled the examination of changes in sedentary time and light-intensity physical activity. Fifteen second epochs were used for both devices. Data were downloaded using manufacturer proprietary software (ActiLife version 6.13, activPAL Professional version 7.2.32) and processed using a customised Excel macro. Twenty minutes of consecutive zeros were considered to be non-wear time [33]. The school timetable was used to extract data for school class periods of interest (lessons in the classroom with the height-adjustable desks and comparison lessons).

As there are no public health guidelines regarding maximum length of sitting bouts to benefit health, a number of bout range frequencies were examined for the *activ*PAL (e.g., 2–5, 5–10, 10–15, and ≥ 15 minutes). For the ActiGraph, sedentary time (< 1.5 METs) was defined as < 100 counts/minute (cpm), which has been found to be a good indicator of free-living sitting time in children [14]. Time spent in light-intensity physical activity (1.5–3.99 METs) was defined as 101 to 2220–3000 cpm (upper limit varied based on age of student [34], [35]). For data from relevant periods to be included in the analyses, adolescents needed to have worn the monitors for at least 50% of the period [14], [36] on a day they were recorded as being present at school. For those with valid data, the proportion of lesson time in which the monitor was worn was high in both the height-adjustable (*activ*PAL 99% and ActiGraph 98% of the period) and comparison (*activ*PAL 97% and ActiGraph 93% of the period) classrooms. The proportion of adolescents with valid data in the height-adjustable and comparison classroom was high for the *activ*PAL (79% and 81%, respectively) and the ActiGraph (77% and 86%, respectively), and over half the sample had valid data for both the height-adjustable and comparison lesson for the *activ*PAL (65%) and ActiGraph (67%). For each outcome variable (Table 1), time spent sitting/sedentary and standing/light-intensity physical activity and the frequency of bouts (sitting and standing) were summed and divided by the number of lessons where valid data were obtained. For inferential purposes, variables were standardised for device wear time by dividing by lesson wear time and then multiplying by total lesson lengths.

### 3.2. Adolescents' perceptions

At the end of seven weeks, adolescents were asked to complete a paper questionnaire that asked whether they would like to continue using the height-adjustable desks during lessons (yes/no) and the perceived impact of the desks on lesson enjoyment, energy/activity levels, muscle pain, and academic behaviours (i.e. ability to complete classwork). Adolescents responded to these items using a 4-point scale ranging from strongly disagree to strongly agree, and responses were dichotomised into agree or disagree. These items and responses are presented in Table 2. Adolescents were also asked to self-report the extent to which they used the desks during classroom lessons (e.g. “I stood up for most of the lesson” “I stood up for some/half the lesson” “I did not stand up during the lessons at all”). These items were asked in relation to morning and afternoon classroom lessons. Adolescents responded using a 4-point scale ranging from strongly disagree to strongly agree, with responses being dichotomised into agree or disagree (see Table 3).

### 3.3. Teachers' perceptions

Teachers were asked to indicate (yes/no) whether they would like to continue to use the height-adjustable desks to deliver lessons where adolescents could stand, and their perceptions of the impact of standing during lessons on adolescents' academic behaviours (e.g. ability learn, distractions; items are listed in Table 4). Teachers responded using a 4-point scale ranging from strongly disagree to strongly agree, and responses were dichotomised into agree or disagree.

## 4. Statistical analyses

Data were analysed using the Statistical Package for the Social Sciences (SPSS) (version 22; IBM Corp, 2012) and STATA SE (version 13; StataCorp LP, 2012). Adolescents' and teachers' responses regarding the feasibility and use of the height-adjustable desks are reported descriptively (percentage; Tables 2 and 4). Differences in self-reported standing between morning and afternoon classes in the intervention classroom were compared using McNemar tests. Chi-square tests of independence were used to examine if the sample with valid *activ*PAL and ActiGraph data from both the height-adjustable and comparison lesson differed from the full sample in regard to age, sex, and year level. As the classroom lessons differed in length, time spent sitting/sedentary and standing/light-intensity physical activity time was quantified as the mean proportion of the lesson time spent in the different behaviours (Table 1). To examine differences in outcome variables in the classroom with the height-adjustable desks and the traditional classrooms, paired sample t-tests were used and effect sizes calculated (Cohen's *d*) [37]. Statistical significance was set at *p* < 0.05.

## 5. Results

Overall, 41 adolescents in Years 7, 9 and 10 completed the survey (51% male, mean age 13.7 ± 1.4 years, age range 12 to 16 years). The sample with valid *activ*PAL data included 28 adolescents (54% male, mean age 13.7 ± 1.4 years) and the sample with valid ActiGraph data included 29 adolescents (55% male, mean age 13.8 ± 1.5). Chi-square tests for independence indicated no significant differences between those with (i) valid *activ*PAL and (ii) ActiGraph data (for both classroom of interest) and the full sample in regard to sex, year level, and subject. Ten teachers (50% male, predominantly aged between 30–39 years) completed the survey.

### 5.1. Impact of height-adjustable desks on adolescents' sitting, sitting bouts, standing, light-intensity physical activity, and academic behaviours

Table 1 displays the results from the paired-sample t-tests examining the differences in objectively-assessed outcomes between the two types of classrooms. The effect size (Cohen's *d*) is also presented. When in the classroom with the height-adjustable desks, based on the *activ*PAL data, adolescents spent significantly less time sitting (60% vs 90% of lesson time, *d* = 0.8) and significantly more time standing (30% vs 10% of lesson time, *d* = 0.8) compared to lessons in traditional classrooms. Results also indicated more frequent short sitting bouts (between 5–10 minutes), fewer longer sitting bouts (15+ minutes), and longer standing bouts (10+ minutes) in the intervention compared to the traditional classroom. No other significant results were observed.

**Table 1.** Means (standard deviations [SD]), paired-sample t-test results comparing behaviour between classrooms and effect sizes (Cohen's *d*) (n = 28)

	Height-adjustable desk classroom	Traditional classrooms	p-value	Cohen's *d*
**Overall posture/behaviour (% proportion/lesson, SD)**				
Sitting^a^	**63 (35)**	**88 (10)**	**< 0.001**	**0.87**
Sedentary time^b^	82 (10)	81 (10)	0.342	0.18
Standing^a^	**32 (32)**	**8 (8)**	**< 0.001**	**0.81**
Light-intensity physical activity^b^	15 (10)	13 (8)	0.273	0.21
**Sitting bouts^a^ (number/lesson, SD)**				
2–5 minutes	0.96 (1.35)	0.82 (1.13)	0.565	0.14
5–10 minutes	**0.84 (0.98)**	**0.23 (0.41)**	**0.005**	**0.79**
10–15 minutes	0.34 (0.55)	0.27 (0.42)	0.529	0.12
15mins+	**0.64 (0.83)**	**1.07 (0.57)**	**0.027**	**0.57**
**Standing bouts^a^ (number/lesson (SD))**				
2–5 minutes	1.42 (1.64)	0.75 (0.99)	0.214	0.26
5–10 minutes	0.52 (1.06)	0.21 (0.46)	0.109	0.35
10–15 minutes	**0.20 (0.39)**	**0.00 (0.00)**	**0.013**	**0.70**
15mins+	**0.30 (0.51)**	**0.02 (0.09)**	**0.009**	**0.71**

Note: ^a^measured by the *activ*PAL; ^b^measured by the ActiGraph. Significant (*p* < 0.05) results in bold.

### 5.2. Adolescents' perceptions of the height-adjustable desks

The majority of adolescents reported that they would like to continue using the height-adjustable desks during classroom lessons, and around half reported enjoying lessons more since the desks were introduced (Table 2). While standing during lessons, most adolescents reported they worked well, and approximately one-third reported finding it difficult to pay attention and that they were easily distracted.

**Table 2.** Adolescents' perceptions of standing during classroom lessons (n = 39)

Questions:	Agree (%)
Like to continue using height-adjustable desks to stand during classroom lessons	70
**When standing during lessons ‘I’…**	
Enjoyed lessons more	54
Concentrated better on doing my work	44
Worked well during lessons	69
Found it difficult to pay attention during lessons	28
Was easily distracted	36
Felt more energetic across the day	46
Was too tired to be active after school	18
Got pain in my legs or back while standing during lessons	51

A significantly greater proportion of adolescents reported that they did not stand up during lessons in the intervention classroom in the afternoon compared to morning classes (Table 3).

**Table 3.** Percentage of adolescents who reported standing durations in morning and afternoon classes (n = 39)

	Morning lessons Agree (%)	Afternoon lessons Agree (%)	p-value^a^
Stood for most of lessons	26	14	0.38
Stood for some/half of lesson	47	42	0.73
Did not stand up at all during lesson	**23**	**40**	**0.03**

^a^p-value from McNemar's test comparing difference between adolescents self-reported standingduring morning and afternoon lessons, significant results in bold

### 5.3. Teachers' perceptions of the height-adjustable desks

Most teachers reported wanting to continue to use the height-adjustable desk during lessons (Table 4). Few teachers agreed that standing during lessons changed adolescents' ability to learn/complete classwork, few reported that using the height-adjustable desks had a negative influence on adolescents' ability to work effectively, and only 14% reported the desks resulted in loss of concentration for the adolescents. No teachers reported that adolescents were too disruptive when using the desks to stand.

**Table 4.** Teachers' perceptions of adolescents standing during lessons (n = 10)

Questions:	Agree (%)
Continue teaching with the height-adjustable desks	71
**Adolescent standing during lessons…**	
Negatively influenced ability to work effectively	14
Results in loss of concentration	14
Increase ability to complete tasks	29
Were too disruptive	0

## 6. Discussion

This pilot study examined differences in adolescents' objectively-measured sitting/sedentary, standing/light-intensity physical activity and sitting and standing bouts between classrooms with traditional desks and height-adjustable desks. It also evaluated the feasibility of replacing traditional classroom desks with height-adjustable desks from the perspective of teachers and adolescent students using the secondary school classroom. It provides some of the first published evidence internationally that implementing height-adjustable desks in a secondary school classroom is feasible and may reduce the time adolescents spend sitting in class compared to traditional classrooms. Significant differences between classrooms were observed for objectively-measured behaviours, with adolescents spending 24% more time standing and 25% less time sitting (and fewer sustained sitting bouts of over 15 minutes) when in the height-adjustable compared to the traditional classroom. Encouragingly, most adolescents and teachers reported wanting to continue to use the height-adjustable desks to stand during classroom lessons, and they reported few adverse impacts on ability to complete classwork.

### 6.1. Classroom sitting and standing

The present study found that during classroom lessons with height-adjustable desks, compared to traditional desks, adolescents spent less time (approximately 20 minutes) sitting and more time (approximately 17 minutes) standing. However, comparisons with past research is difficult since most have examined the primary (elementary) school setting. Primary school children remain in the same classroom for most lessons throughout the year enabling changes in sitting across the whole school day to be examined [22], [38] using a variety of objective measures of behaviour [24], [29]. A study of the impact of height-adjustable desks among two samples of primary school children in the UK and Australia found a similar proportion of classroom lesson time was spent sitting (59–62%) and standing (24–26%) [15] compared to what the current study found in the intervention classroom (sitting 63%; standing 32%). If all classrooms had height-adjustable desks installed the potential reduction in sitting across a school day could be as much as one hour and 40 minutes, based on the 20-minute difference in sitting per lesson seen in the current study multiplied by five lessons per day.

The results of the present study also indicated that height-adjustable desks may contribute to reducing prolonged periods of sitting. A novel aspect of the study was the examination of changes in the length of bouts of sitting and standing during classroom lessons equipped with height-adjustable desks. Fewer sustained bouts of sitting were accumulated in the height-adjustable desk classroom compared to the traditional classroom. While these effects were smaller in magnitude (Cohen's *d* 0.70) than those observed for overall sitting time (Cohen's *d* 0.87), this is promising in light of research suggesting the school context, compared to after school context, is associated with more frequent prolonged sedentary bouts (> 20 minutes) [18]. In addition, recent research has indicated that sedentary time accumulated in bouts more than 15–20 minutes may be related to adverse cardio-metabolic outcomes in adults [35], [39], [40], and bouts of 5–10 minutes have been negatively associated with inflammatory markers in children [5]. While there is no dose-response evidence available regarding reducing and breaking up sitting and children's and adolescents' health outcomes, the Australian Government [13] recommends breaking up and reducing the volume of sitting throughout the day. These results indicate that the installation of height-adjustable desks in secondary schools could make a significant contribution to meeting these public health guidelines.

While these results are positive, it is important to consider potential adverse outcomes. Prolonged periods of standing may induce musculoskeletal pain and discomfort [41]. Half the adolescents in the current study reported feeling pain in their legs or back while standing during lessons. While past research has found no difference in musculoskeletal discomfort with stand-biased desks among children (beyond normal aches and pains) [38] and a decrease in trunk tension among adolescents with height-adjustable desks [27], differences in exposure levels to desks and time taken to acclimatise to the desks is likely to influence outcomes (exposure time to desks varied among studies from four weeks [38] to two years [27]). When examining the musculoskeletal impact of classroom sitting and standing, it is important for future research to consider developmental issues during adolescence (a time naturally associated with growth aches and pain). The impact of prolonged periods of standing on different muscle groups, and how to acclimatise adolescents to increased standing is another area in need of further investigation [27], [28]. As the current study did not provide guidance around what adolescents ‘should’ do in regard to sitting/standing, the results suggest that adolescents stood more during morning versus afternoon classes, and most engaged in short standing bouts between 2-to-5 minutes. These results provides insight for future interventions, specifically that the provision of standing targets of 2-to-5 minutes in morning classes may be a feasible starting point to accustom students to gradually increase their standing time as a means to reduce/break-up classroom sitting. However, it is important to emphasise that ergonomically, frequent postural transitions are likely to be more feasible and better for adolescents' musculoskeletal health than expecting them to stand for the entire school day [19].

### 6.2. Feasibility

A majority of adolescents (70%) and teachers (71%) reported wanting to continue to use the height-adjustable desks for learning and teaching during classroom lessons, suggesting the desks were acceptable and liked within the secondary school setting. This is consistent with previous research that has found most students and teachers were satisfied and supportive of height-adjustable/standing workstations in primary [22], [38] and secondary school settings [27]. A novel aspect of the study was providing a height-adjustable desk for the teachers to use, with the majority reporting that they wanted to continue to use it. Future research may benefit from examining the potential influence of role modelling and how this could further reduce and break-up adolescents' classroom sitting.

As a result of standing during classroom lessons, half the students reported feeling more energetic across the day, with only a few (18%) reporting that there were too tired to be active after school. These findings are consistent with the activity synergy hypothesis which proposes that engagement in an active behaviour during one part of the day may increase physical activity in other parts of the day [42]. Only a few adolescents indicated standing during lessons made them feel tired (across the day and after school). However, a greater percentage of students reported they did not stand up during afternoon lessons compared with morning lessons. This may be due to general daily tiredness and provides insight for future interventions with a feasible starting point standing more in the mornings than afternoons. While the current study did not examine physical activity compensation after school as a result of using the desks, recent research with adults found increases in standing/light-intensity physical activity at work were associated with increases in sitting and decreases in physical activity outside of work to compensate for these changes [43]. Further research is needed to determine whether adolescents who use height-adjustable desks during school hours then compensate for this by decreasing their activity after school, or whether this results in higher levels of activity after school.

While the majority of adolescents reported working well with the desks, a third reported difficulties paying attention and becoming distracted while standing during classroom lessons. Encouragingly, few teachers reported negative impacts on academic behaviours (e.g. distractions, loss in concentration). Past research in the secondary school setting found after 24 months using height-adjustable desks adolescents had higher grades relative to adolescents who did not use these desks [27], and a recent pre-post pilot study among high school students found after using sit-stand desks for 28 weeks students had significant improvements in executive function and working memory capabilities [31]. It is possible that the reported difficulties in concentration and attention in the current study may relate to the low levels of exposure to the height-adjustable desks, as students only had 1–2 lessons in this classroom per week (which suggests lessons in this room were quite novel). It may also be that some students take longer to adjust to standing during lessons, and this type of option in class may not suit all adolescents. In addition, it is not known if the perceived distractions were due to the adolescents' own use of the desks or due to others raising and lowering the desks (or possibly both). Greater exposure may be needed for students to adjust and become accustomed to working while standing.

From the primary school setting, whilst the results have been mixed (e.g., teacher-rated improvements in attention/focus [30], no change in students' active, passive, off task behaviour, and academic behaviour [23]), they generally indicate no adverse effects associated with standing. Overall, the current study and past research suggest it may be possible to change the classroom environment within schools to decrease sitting without jeopardising student academic performance/engagement, however, the effect on academic behaviours in secondary schools when standing needs to be further explored particularly that associated with more frequent and regular use of height-adjustable desks relative to control students during lessons with traditional desks. Showing that height-adjustable desks in the classroom do not negatively affect attention, learning or academic outcomes, and in fact may be of benefit, is important for uptake of these desks in the education system. Further research is needed using more objective and systematic methods of academic behaviours, academic grades, and cognitive ability using study designs with control groups to account for learning and development effects (i.e. gains made in academic ability associated with normal academic and developmental progression over time) [19], [20]. Furthermore, research must be aware of how potential unintended consequences on academic behaviours associated with standing and breaking up sitting may transfer to academic grades, these aspects should be monitored as standard in future interventions.

### 6.3. Limitations

A number of limitations warrant attention. The height-adjustable desks were installed in only one classroom in one school with adolescents only having 1–2 lessons per week in that classroom (low exposure), and the within-subject comparison at one time point were limitations. Furthermore, the survey items examining time of day standing, perceived acceptability and academic behaviours were exploratory. As such, the interpretation of these items is limited. Future research should use items that are balanced (between positive and negative views) and that also include questions about lessons in traditional classrooms. Participation rates among adolescents and teachers were low, which may limit the generalisability of the results. Further research with larger representative adolescent samples is needed.

Unlike the significant results with the *activ*PAL, no significant differences between the traditional and height-adjustable desk classrooms were observed for sedentary or light-intensity physical activity time using the ActiGraph accelerometer. The ActiGraph accelerometer has a limited ability to distinguish between sitting and standing when movement is minimal [14], [44], suggesting that using an objective measure that can directly assess seated posture and changes from sitting to standing is important for determining intervention effects.

Future research should adopt a stronger experimental design (e.g. cluster randomised controlled trial) and use objective measures of classroom behaviour, energy levels, academic markers, anthropometric, and musculoskeletal pain/discomfort. Future research would also benefit from examining the feasibility and impact of messages relating to reducing sitting and changing postures (e.g. frequency of sitting breaks, length of standing bouts). Incorporating behavioural messages and strategies in addition to the provision of height-adjustable desks may have additional benefits for reducing both sustained sitting and standing bouts and minimising potential muscular discomfort [19].

### 6.4. Conclusions

This pilot study is one of the first to examine differences in adolescents' sitting and standing in a secondary school classroom with height-adjustable desks compared to traditional classrooms, and the feasibility of these desks from students' and teachers' perspectives. Results from this pilot study provide preliminary data that is consistent with research in the primary school setting in that height-adjustable desks in secondary school classrooms may have the potential to improve adolescents' health through reducing sitting [20]. Given previous research has shown that sedentary behaviour among children and adolescents tracks into adulthood, establishing healthy habits in the classroom may transfer to adulthood and the next generation of workers. Providing adolescents with the choice to sit or stand during classroom lessons may assist in changing norms around sitting for study and work pursuits and raise expectations that could help drive cultural change within workplaces in the future [7].
